# Prevalence and determinants of stillbirth among women attended deliveries in Aksum General Hospital: a facility based cross-sectional study

**DOI:** 10.1186/s13104-019-4397-7

**Published:** 2019-07-01

**Authors:** Tesfay Berhe, Hailay Gebreyesus, Haftom Teklay

**Affiliations:** 1 Department of Public Health, College of Health Sciences, Aksum University, P. O. Box: 298, Aksum, Ethiopia; 2 JSI/L10K, Mekelle, Ethiopia

**Keywords:** Stillbirth, Delivered mothers, Aksum, Ethiopia

## Abstract

**Objective:**

In Ethiopia skilled deliveries are increasing but stillbirth is not reducing as required. However; there are limited numbers of up to date studies done related to stillbirth in the study area. Therefore this was aimed to assess the prevalence and determinants of stillbirth using facility based cross-sectional study among women attended deliveries at Aksum General Hospital in 2018. Systematic random sampling method was used to select 573 study participants from the deliveries attended during the study period. The data was entered into Epi-data version 3.1 and exported to Statistical Package for Social Science version 21 for analysis. Bivariate and multivariable logistic regression analysis were conducted to identify significant predictors and strength of association was measured based on adjusted odds ratio with 95% confidence level and statistical significance was declared at p-value less than 0.05.

**Results:**

The prevalence of stillbirth was 3.68% in this study area. Maternal age 20–35 (AOR = 0.25; 95% CI (0.08, 0.80)), not using partograph (AOR = 8.66; 95% CI (2.88, 26.10)) and gestational age < 37 weeks (AOR = 3.86; 95% CI (1.27, 11.69)) were the independent factors affecting the stillbirth.

## Introduction

Stillbirth is one of the adverse births outcomes and represent major problem in both developing and developed countries [1]. Developing countries are remained as the major contributors of stillbirth in which Ethiopia is among those in the higher prevalence [2–5]. It is also the reflections of quality of care during pregnancy and child birth; skilled deliveries are increasing but stillbirth is not well reducing as required in the developing world including Ethiopia [3–6].

There were 2.6 million stillbirths in 2015 worldwide [6–8]. Stillbirth due to intra-partum loss is higher in developing than developed countries which is 59% and 10% respectively. It has been noted that 99% of these deaths occur in the low and middle income nations [9]. It is said that about 66% of the worldwide stillbirths is contributed by developing nations like India, Pakistan, Nigeria, China, Demographic Republic of Congo, Ethiopia, Bangladesh, Indonesia, Tanzania and Afghanistan. However, the stillbirth rate has come down by 19.4% from 2000 (1.02 million) to 2015 (2.6 million) with various interventions globally [8, 10].

In Ethiopia the stillbirth rate was 10.4, 16.9 and 11 per 1000 births in 2000, 2011 and 2016 [11, 12]. This shows that the progress on reducing stillbirth rate was poor. Under-5 mortality rates are reducing in many countries and Ethiopia also achieved the millennium development goal on child mortality reduction [13, 14]. The government of Ethiopia had been implementing different effective programs so as to improve maternal and child health issues under the Health Extension Package like; immunization, community based nutrition, integrated management of Community Case Management on childhood illness and capacity building health professionals so as to improve the quality of service during pregnancy like; antenatal and delivery care [15]. However, the outcome during pregnancy and delivery periods is a major challenge in realizing the seated goal in the Sustainable Development Goals (SDGs) i.e. stillbirth and neonatal mortality shown less progress in Ethiopia [13, 14].

Thus, to reduce these problems, identifying the prevalence and factors that affect stillbirth in a setup is critically important. However, setup based information in the study area is limited. The aim of this study was to assess the prevalence and determinants of stillbirth among women attended deliveries at Aksum general hospital, Tigray, Northern Ethiopia in 2018.

## Main text

### Methods

#### Study area and period

The study was conducted from January to March 30, 2018 in Aksum town which is 1010 km away from north of Addis Ababa and 250 km far from Aksum (capital city of Tigray region). According to the town administrative office report 2019, the town has about 75,842 total populations, of which about 51% are female and 49% are male. Totally about 1759 deliveries were attended at Aksum general hospital in 2017. Administratively, the town is divided into five kebeles (the smallest administrative units). In the town there are two health centers, one general hospital, one referral hospital and four private clinics.

#### Study design and populations

This study was conducted by using facility based quantitative cross-sectional study design. Mothers who gave birth at Aksum general hospital who were randomly selected included in the study and those critically sick mothers who cannot respond during data collection were excluded from the study.

#### Sample size and sampling techniques

The sample size was determined by using single population proportion formula (i.e. n = z^2^p (1 − p)/d^2^) using the following assumption: proportion of stillbirth (p) = 9.6%, [16], margin of error (d) = 3%, 95% confidence level, design effect 2, using correction formula and 10% for possible non response rate. So the final sample size was 573. The first mother from the delivery register was identified using a lottery method. Then systematic random sampling technique was used to select study participants from the register within the general hospital.

#### Operational definitions

History of poor obstetric outcome: Mothers who had history of LBW, preterm birth, stillbirth, perinatal death or abortion.

Stillbirth: Stillbirth is a baby born with no signs of life at or after 28 weeks’ gestation.

#### Data collection tool

A structured and quantitative interviewer administered questionnaire was adopted from WHO and different literatures [17, 18]. Data was collected from mothers using structured and pretested questionnaire. First the questionnaire was prepared in English and translated to local language Tigrigna and translated back to English to observe its consistency. Finally, the questionnaire was pre tested on 5% of mothers before the actual data collection in Adwa general hospital; correction and modification were done based on the gap identified during the pre-test interview.

#### Data collection technique and quality control

Interviewer-administered questionnaire were used for the data collection method. The data was collected by five clinical nurses and supervised by two BSc midwives professionals and the principal investigator. Training was given for data collectors and supervisors on the aim of the research, content of the questionnaire and how to conduct. Data on stillbirth was collected after the delivered mother was stabilized and before discharged to home. Additionally medical record of the mother was reviewed for the remaining data. The collected data was checked every day by supervisors and principal investigator for its completeness and consistency.

#### Data processing and analysis

The data was entered, cleaned and coded using Epi-data version 3.1 and was analyzed using SPSS version 21. Descriptive statistics was used to present categorical data using frequency tables and bar graph. The association between dependent and each independent factor was analyzed using bivariate logistic regression model with crude odds ratio and 95% confidence interval. Factors with p-value < 0.25 were further analyzed using multivariable logistic regression analysis to determine factors associated with stillbirth. Finally p-value at < 0.05 was used to declare the significant associated factors with the outcome variable (stillbirth). Hosmer and Lemeshow goodness-of-fitness was also used to test model fitness. In addition multi-collinearity between independent variables was also checked.

### Results

#### Socio-demographic and economic characteristics of respondents

A total of 570 mothers were interviewed which gives 99.5% of response rate. Four hundred forty-three (77.7%) of the study participants were in the age group of 20–34 years and 547 (96%) were Orthodox followers. Most of the respondents (n = 542; 95.1%) were Tigrean by ethnicity and 550 (96.5%) were married. Four hundred eighty-nine (86%) of the study participants were lived with family size of below five and two hundred thirty-seven (41.6%) had monthly income of 2500–5000 Ethiopian birr (Table 1).

**Table 1:** Socio-demographic characteristics of respondents assessed on stillbirth in Aksum hospital, Tigray, Ethiopia, 2018 (n = 570)

Variables	Number	Percent
Maternal age in years		
< 20	68	11.9
20–34	443	77.7
35+	59	10.4
Residence		
Urban	531	93.2
Rural	39	6.8
Marital status		
Single	20	3.5
Married	550	96.5
Religion		
Orthodox	547	96
Muslim	23	4
Ethnicity		
Tigrean	542	95.1
Others^a^	28	4.9
Maternal educational status		
Illiterate	56	9.8
Elementary	177	31.1
Secondary and above	337	59.1
Maternal occupation		
Housewife	345	60.5
Daily laborer	25	4.4
Employed	87	15.3
Merchant	82	14.4
Student	31	5.4
Monthly income		
< 2500 birr	126	22.1
2500–5000	237	41.6
> 5000	207	36.3
Family size		
< 5	489	85.8
≥ 5	81	14.2

NB: Others^a^ Amhara and Afar

#### Gynecological and obstetrical, and newborn related characteristics of respondents

From the total respondents 372 (65.3%) were multipara, and 234 (62.9%) gave birth between 2 and 5 years from previous pregnancy. Most of the mothers 469 (82.3%) were received ANC four times and above and 459 (80.7%) initiated ANC at first trimester of the current pregnancy. Most of the pregnancies (n = 529; 92.8%) were planned and 556 (97.5%) mothers were not faced any pregnancy related complication during current pregnancy. Regarding history of poor obstetric outcome, 115 (20.2%) of participants had faced any history of poor obstetric outcome in their previous pregnancies. The finding of this study showed that the prevalence of stillbirth was 3.68% (Table 2).

**Table 2:** Gynecological and obstetric, and newborn characteristics of respondents Aksum hospital, Tigray, Ethiopia, 2018 (n = 570)

Variables	Frequency	Percent
ANC visit		
Less than four	101	17.7
Four and above	469	82.3
Initiation of ANC		
First trimester	459	80.7
Second trimester	105	18.4
Third trimester	5	0.9
Parity		
Prim-gravid	198	34.7
Multi gravid	372	65.3
Pregnancy type		
Singleton	563	98.8
Multiple	7	1.2
Mode of delivery		
SVD	488	85.6
Assisted delivery	20	3.5
Cesarean section	62	10.9
Birth interval (years)		
< 2	46	12.4
2–5	234	62.9
> 5	92	24.7
Pregnancy status		
Planned	529	92.8
Unplanned	41	7.2
Partograph use		
Yes	500	87.7
No	70	12.3
Labor status		
Spontaneous	528	92.6
Induced	42	7.4
History of poor obstetric outcome		
Yes	115	20.2
No	455	79.8
Pregnancy related complication		
Yes	14	2.5
No	556	97.5
Hemoglobin level (mg/dl)		
< 11	26	4.6
≥ 11	544	95.4
Labor complication		
Yes	68	11.9
No	502	88.1
Birth outcome		
Stillbirth	21	3.68
Live birth	549	96.32

#### Factors associated with stillbirth

According the binary logistic regression maternal age, gestational age, antenatal care, counseling on additional diet during pregnancy, additional diet during pregnancy and partograph use were showed significant association with stillbirth at p-value < 0.05.

In multiple variable logistic regressions analysis, after controlling all other variables ante natal care, counseling on additional diet during pregnancy and additional diet during pregnancy were not significantly associated with stillbirth. But maternal age, gestational age and partograph use had shown significant association with stillbirth.

Newborn infant from mothers aged 20–35 were 4 times less likely to be stillbirth as compared to mothers in the age group ≥ 35 (AOR = 0.25; 95% CI (0.08, 0.80)) and mothers who didn’t monitored with partograph were almost 9 times more likely to give stillbirth compared these monitored by partograph (AOR = 8.66; 95% CI (2.88, 26.10)). Regarding gestational age newborn infant who were born in < 37 weeks of gestational age were almost 4 times more likely to be stillbirths compared to these who born ≥ 37 weeks of gestational age (AOR = 3.86; 95% CI (1.27, 11.69)) (Table 3).

**Table 3:** Factors associated with stillbirth among deliveries at Aksum hospital, Tigray, Northern Ethiopia, 2017 (n = 570)390

Variables	Stillbirth		COR (CI-95%)	AOR (CI-95%)	p-value
	Yes n (%)	No n (%)			
Maternal age					
< 20	3 (4.4)	65 (95.6)	0.41 (0.10–1.71)	0.36 (0.07–1.89)	0.28
20–35	12 (2.7)	431 (97.3)	0.25 (0.09–0.68)	0.25 (0.08–0.80)*	0.023
≥ 35	6 (10.2)	53 (89.8)	1	1	
ANC visit					
Less than four	9 (8.9)	92 (91.1)	1	1	
Four and above	12 (2.6)	457 (97.4)	0.27 (0.11–0.66)	0.41 (0.13–1.30)	0.116
Arrival at HF					
Before onset of labor	5 (6)	78 (94)	1.887 (0.67–5.30)	1.51 (0.39–5.91)	
After onset of labor	16 (3.3)	471 (96.7)	1	1	
Baby’s sex					
Male	15 (4.9)	292 (95.1)	2.2 (0.84–5.76)	1.657 (0.58–4.75)	
Female	6 (2.3)	257 (97.7)	1	1	
Partograph use					
Yes	10 (2)	490 (98)	1	1	
No	11 (15.7)	59 (84.3)	9.14 (3.72–22.42)	8.66 (2.88–26.10)*	0.001
Gestational age					
< 37 weeks	10 (13.9)	62 (86.1)	7.14 (2.91–17.50)	3.86 (1.27–11.69)*	0.011
≥ 37 weeks	11 (2.2)	487 (97.8)	1	1	
Counseling on diet					
Yes	9 (2.3)	389 (97.7)	1	1	
No	12 (7)	160 (93)	3.24 (1.34–7.84)	9.94 (0.80–124.12)	0.085
Additional diet					
Yes	10 (2.5)	385 (97.5)	1	1	
No	11 (6.3)	164 (93.7)	2.58 (1.08–6.20)	0.19 (0.14–2.51)	0.225

*AOR* adjusted odds ratio, *CI* confidence interval, *COR* crude odds ratio

1: referent

*Significant at p-value < 0.05

### Discussion

According the findings of this study prevalence of stillbirth was 3.68%. The prevalence of stillbirth is almost consistent with a study conducted in Nigeria which shows 4% prevalence [19]. Whereas lower than the studies conducted at Shire hospital, Dessie referral hospital, Gondar university hospitals and Ngest Eleni Mohammed memorial general hospital and which shows 9.6%, 14%, 7.1% and 9.8% respectively. The variations between these findings may be attributable to the variations in socioeconomic variations of the study subjects in whom most of our study participants were urban residents which may resulted in improved birth outcome [16, 20–22].

Mothers’ age was significantly associated with stillbirth in this study. Newborn infant from mothers aged 20–35 years were 75% times less likely vulnerable to stillbirth as compared to newborn infants from mothers in the age group ≥ 35 years. This study is consistent with a logistic regression analysis on determinants of stillbirth in Ethiopia, Tanzania and systemic review conducted by Canadian medical association and its licensors [23–25]. The reasons that stillbirth rates increase with maternal age are currently unclear. Even in uncomplicated pregnancies there is increased risk in stillbirth associated with maternal age. In older mothers, stillbirth rate increases as the gestational age increases beyond 37 weeks [26].

Newborn born before 37 weeks of gestational age are with increased risk of stillbirth. This is consistent with study conducted in Tanzania [24]. This association might be due to premature. Immature newborns have less time to grow in the mother’s uterus and they are at risk of being asphyxiated and distressed and lead them to stillbirth [21].

Partograph utilization by service providers was the other strong factor associated with stillbirth. Participants who were not served using partograph were in higher odds of facing stillbirth than their counter parts. Poor partograph utilization may resulted in prolonging second stage of labor which could also resulted in depriving interventions like; cesarean section, augmentation and instrumental delivery [26–28].

### Conclusion

Based on this study stillbirth prevalence was high. Factors like maternal age ≥ 35 years, gestational age < 37 weeks and lack of partograph use during delivery also associated with stillbirth. The determinants identified in this study can be prevented and managed by providing appropriate care during ante-partum and intra-partum period.

## Limitation of the study

This study was conducted within single pubic hospital on limited number of participants (small sample size) where at delivery clinic which means the finding may not be generalizable to the overall Ethiopian delivered mothers in this or other public or private clinics. This cross sectional nature of the data shares the weakness of cross sectional study. It is impossible to draw inferences about the direction of relations among study variables. These data are retrospective and thus are subject to recall bias. Then the currently reported prevalence may under estimate the real prevalence in the ground.

## Data Availability

The datasets in which conclusion taken is available in the form of Microsoft Excel. It is available on requesting.

## Abbreviations

AOR: adjusted odds ratio
BSc: Bachelor of Science
COR: crude odds ratio
EDHS: Ethiopian Demographic and Health Survey
HF: health facility
km: kilometer
LBW: low birth weight
MUAC: Middle Upper Arm Circumference
SPSS: Statistical Package for Social Science
USA: United States of America
WHO: World Health Organization
